# When AI joins the table: evaluating large language model performance in soft tissue sarcoma tumor board decisions

**DOI:** 10.1007/s00432-026-06432-w

**Published:** 2026-02-27

**Authors:** Reza Dehdab, Saif Afat, Fiona Mankertz, Jan Michael Brendel, Nour Maalouf, Sebastian Werner, Andreas Brendlin, Judith Herrmann, Konstantin Nikolaou, Linus D. Kloker, Branko Calukovic, Katrin Benzler, Lars Zender, Christoph K. W. Deinzer

**Affiliations:** 1 Department of Radiology, Tübingen University Hospital, University of Tübingen, Tübingen, Germany; 2 DFG Cluster of Excellence 2180 ‘Image-Guided and Functional Instructed Tumor Therapy’ (iFIT), University of Tübingen, Otfried-Müller-Str. 10, 72076 Tübingen, Germany; 3 Center for Soft Tissue Sarcomas, GIST and Bone Tumors (ZWS) of the University Hospital Tübingen and the Comprehensive Cancer Center (CCC) Tübingen-Stuttgart, Tübingen, Germany; 4 Department of Internal Medicine VIII - Medical Oncology and Pneumology, Medical University Hospital Tübingen, Tübingen, Germany

**Keywords:** Artificial intelligence, Large language models, Multidisciplinary Tumor Boards, Soft tissue sarcoma, Clinical decision support

## Abstract

**Objectives:**

Multidisciplinary tumor boards (MDTs) are critical for the personalized management of soft tissue sarcomas (STS), but they are limited by time, costs, and resource demands. With recent advances in large language models (LLMs) like ChatGPT, there is growing interest in evaluating their potential role in augmenting MDT workflows. This study aimed to assess the clinical performance of ChatGPT-4o in real-world STS cases using predefined evaluation criteria, comparing its treatment suggestions with expert MDT decisions.

**Materials and methods:**

This retrospective study included 152 patients presented to the multidisciplinary sarcoma tumor board. ChatGPT-4o was prompted to generate guideline-based treatment recommendations based on anonymized tumor board registration letters. Outputs were scored by blinded expert reviewers using a five-domain framework: diagnostic modalities, therapeutic modalities, treatment sequencing/timing, chemotherapy regimen, and clinical contextualization. Descriptive statistics and non-parametric ANOVA with post hoc tests assessed performance, including subgroup analysis by sarcoma subtype.

**Results:**

ChatGPT-4o scores were significantly lower than the maximum achievable value of 1.0 across all five criteria (all *p* < 0.0001). Among individual domains, clinical contextualization significantly outperformed all other criteria in pairwise comparisons (all *p* < 0.05). No significant performance differences were observed across sarcoma subtypes (H = 19.74, *p* = 0.138).

**Conclusions:**

ChatGPT-4o demonstrated substantial expert-rated performance in generating tumor board recommendations for soft tissue sarcoma cases, particularly excelling in personalized contextualization. Discrepancies in treatment sequencing and chemotherapy selection highlight the need for expert oversight. These findings support the feasibility of LLM integration into oncology workflows, warranting further refinement toward safe, supportive clinical use.

**Supplementary Information:**

The online version contains supplementary material available at 10.1007/s00432-026-06432-w.

## Introduction

Soft tissue sarcomas (STS) represent a rare and heterogeneous group of malignancies, comprising approximately 1% of all adult cancers. Their clinical management requires a multidisciplinary approach encompassing diagnosis, therapeutic planning, and long-term surveillance (Stiller et al. 2013; Ammo et al. 2025).

Multidisciplinary tumor boards (MDT) have consistently served as pivotal platforms for collaborative decision-making, bringing together expertise from surgical, medical, and radiation oncology, as well as pathology, radiology, and other relevant specialties to address the complexity of STS management (Goker et al. 2024). MDT discussions are essential for delivering a comprehensive assessment of each case and for developing personalized treatment strategies that align with the individual patient's clinical profile (Lamb et al. 2011). However, the implementation of MDTs can be constrained by logistical challenges, including financial costs, time demands, geographic disparities, and potential delays in treatment initiation (Makary 2011; Luchini et al. 2020). These constraints have driven growing interest in the application of artificial intelligence (AI) to support or optimize multidisciplinary decision-making (Zhao et al. 2020; Park and Chae 2024).

Recent advances in AI, particularly in the field of natural language processing (NLP), have led to the development of powerful large language models (LLMs), such as the Generative Pretrained Transformer (GPT) series by OpenAI. These models, including ChatGPT, can understand and generate human-like text, enabling applications ranging from automated content creation to simulated medical consultations and health information delivery (Ali et al. 2023). A defining characteristic of LLMs is their capacity to perform a wide range of tasks with minimal or no task-specific training, enabled by few-shot and zero-shot learning (Dehdab et al. 2024). These advances have created opportunities to use LLMs in MDT settings (Sorin et al. 2023a).

To date, the use of AI in tumor board settings has been examined in only a limited number of studies, each addressing different cancer types (Aghamaliyev et al. 2024; Schmidl et al. 2024; Sorin et al. 2023b; Erdat et al. 2025). Initial findings suggest that models such as ChatGPT may offer potential support within MDT settings. However, these studies also highlight current limitations, indicating that LLMs are not yet sufficiently mature for consistent and reliable use in clinical decision-making. While initial efforts have explored AI in tumor boards across various cancer types, its application in the sarcoma setting remains underexplored. Existing investigations have included only a limited number of cases and have primarily relied on subjective assessments, such as Likert scale ratings, rather than predefined evaluation criteria (Ammo et al. 2025).

The aim of this study was to evaluate ChatGPT-4o in real-world sarcoma cases using predefined clinical criteria. The objective was to examine the extent to which ChatGPT-4o’s treatment suggestions correspond to expert recommendations from experienced multidisciplinary oncology teams. This evaluation may help define the potential role of large language models as supportive tools in oncology, particularly for treatment planning and decision-making, while also providing a basis for future development of AI-assisted clinical workflows.

## Materials and methods

### Study design and ethical approval

This study was designed as a retrospective analysis and conducted at the University Hospital Tübingen. Ethical approval was granted by the Institutional Review Board of the Faculty of Medicine, Eberhard Karls University Tübingen, and the University Hospital Tübingen (reference no. 418/2024BO2). Given the retrospective design and the use of anonymized clinical data, the need for informed consent was waived. The study was carried out in accordance with the Declaration of Helsinki.

### Patient selection

**Fig. 1:**
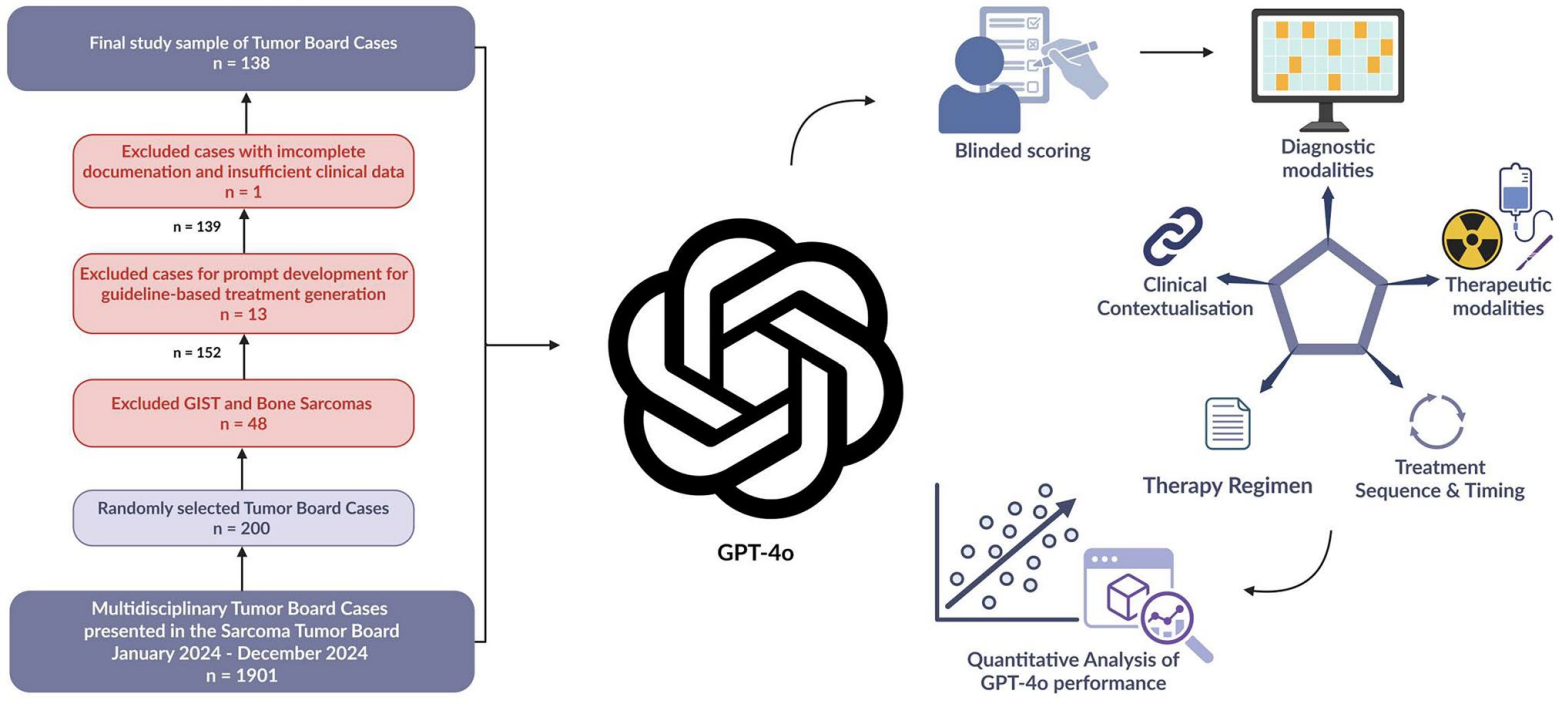
Schematic representation of the study workflow

We retrospectively reviewed all patients who were presented in the multidisciplinary tumor board of the Sarcoma Center of the University Hospital Tübingen and the Comprehensive Cancer Center (CCC) Tübingen-Stuttgart between January and December 2024. From the total tumor board database, a random subset of cases was selected for analysis. Gastrointestinal stromal tumors (GIST) and bone sarcomas were subsequently excluded due to their distinct diagnostic and treatment workflows. Eligible cases required complete documentation of tumor board recommendations and sufficient clinical data. Patients were excluded if key information was missing, including the absence of a documented tumor board recommendation, unstructured or non-standardized referral letters, or insufficient clinical data necessary for evaluation. A schematic overview of the study design is presented in Fig. 1 (Lauer 2025).

## Evaluation framework

To assess the quality and clinical relevance of LLM-based treatment suggestions, we developed a 5-point scoring system based on predefined clinical decision criteria. Each criterion was independently scored by two sarcoma expert reviewers in a blinded fashion, based on how well the model’s suggestions aligned with the actual tumor board recommendations. Discrepancies were resolved by consensus and the final scores were used as the reference standard for analysis.

The following criteria were applied:

Criterion 1: Diagnostic Modalities.

Score: 0–2 points (only assessed if diagnostics were recommended).

- 0 points: The recommended diagnostic test is clearly incorrect or contraindicated. Example: Recommending a chest X-ray instead of a CT Thorax for metastasis staging.
- 1 point: The general direction is appropriate (e.g., a CT is recommended), but critical components are missing or unnecessary tests are added without justification. Example: Recommending CT Thorax and Abdomen, but omitting CT Pelvis or failing to suggest MRI for local staging.
- 2 points: All recommended diagnostic modalities match those from the tumor board. The selection reflects a complete and appropriate staging or follow-up workup. Example: CT of thorax, abdomen, and pelvis + MRI of the affected extremity for a high-grade sarcoma.

Criterion 2: Therapeutic Modalities.

Score: 0 – 2 points.

- 0 points: The proposed treatment is inappropriate or contradicts guidelines.
  Example: Surveillance in a patient with a resectable high-grade sarcoma.
- 1 point: A key therapeutic modality is correctly recommended (e.g., surgery), but others are missing or unjustified therapies are added.
  Example: Recommending surgery but omitting neoadjuvant radiotherapy in a large myxoid liposarcoma.
- 2 points: All necessary therapeutic modalities (e.g., surgery + chemo + radiotherapy) are recommended in line with the tumor board, and their use is well-reasoned.

Criterion 3: Treatment Sequence and Timing.

Score: 0–1 point.

- 0 points: The sequence is inappropriate and contradicts clinical practice.
  Example: Suggesting surgery before neoadjuvant treatment in a large, high-grade tumor.
- 1 point: The sequence is either correct or at least acceptable and consistent with tumor board recommendations.
  Example: Neoadjuvant chemotherapy → neoadjuvant radiotherapy → surgery.

Criterion 4: Chemotherapy Regimen Appropriateness.

Score: 0–1 point (only assessed if chemotherapy was recommended).

- 0 points: The LLM proposes an incorrect or non-standard chemotherapy regimen.
  Example: Recommending Nivolumab/Ipilimumab in a case of metastatic soft tissue sarcoma as first-line therapy.
- 1 point: A guideline-based and clinically appropriate regimen is proposed, including drug type.
  Example: Doxorubicin + Ifosfamide for a metastatic high-grade soft tissue sarcoma.

Criterion 5: Clinical Contextualization and Individualization.

Score: 0–2 points.

- 0 points: No consideration of previous therapies, comorbidities, or contraindications.
  Example: Recommending further Doxorubicin after the patient already reached maximum lifetime dose.
- 1 point: The clinical context is mentioned but only partially or incorrectly applied.
  Example: Acknowledging prior surgery but failing to consider toxicity profile in ongoing treatment.
- 2 points: Prior treatments, patient-specific factors (e.g., frailty, toxicity), and treatment history are thoughtfully incorporated.
  Example: Recommending second-line therapy due to known Doxorubicin intolerance or resistance.

In addition, all model outputs were screened for error types by the two sarcoma experts. This included hallucination, defined as the fabrication of clinical facts not present in the input, and confabulation, defined as the generation of fluent, plausible-sounding but factually incorrect outputs. Discrepancies in classification were resolved by consensus.

### Prompting

**Fig. 2:**
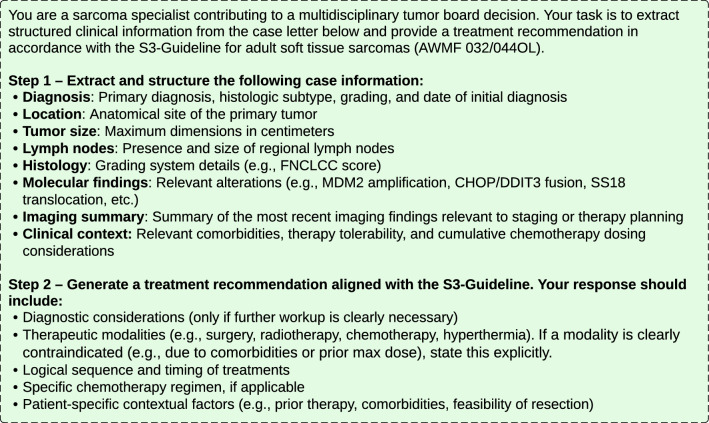
Developed text prompt for ChatGPT to generate guideline-based treatment recommendations

To design and refine a structured prompt for generating guideline-based treatment recommendations for adult soft tissue sarcomas, a total of 13 tumor board registration letters were reviewed by R.D to design and refine a structured prompt for generating S3-guideline-based treatment recommendations for adult soft tissue sarcomas using LLM (Fig. 2). These cases, which were excluded from the primary evaluation cohort, included finalized tumor board decisions, allowing the prompt logic to be tested and adjusted by comparing LLM-generated outputs with actual clinical recommendations. The selection aimed to reflect a representative spectrum of disease stages, histological subtypes, and documentation styles, thereby increasing the robustness and generalizability of the prompt.

The finalized prompt consisted of two sequential tasks. The first part instructed the GPT-4o to extract structured clinical information from the free-text tumor board registration letters. Extraction categories included: diagnosis details (histology, grade, and location), tumor dimensions, relevant molecular alterations (e.g., MDM2 amplification, CHOP/DDIT3 translocation), chronological clinical events, metastatic spread, prior treatments and outcomes, and contextual clinical considerations such as comorbidities, tolerability, or cumulative chemotherapy dosing.

The second part of the prompt directed the model to instruct a guideline-compliant treatment recommendation in accordance with the S3-Guideline for adult soft tissue sarcomas (AWMF 032/044OL) (Leitlinienregister 2025). The model was directed to consider key components including diagnostic considerations, therapeutic modalities, sequencing and timing of treatments, chemotherapy regimens where applicable, and patient-specific contextual factors such as prior therapies or comorbidities. The model was also instructed to explicitly mention contraindications where relevant and to ensure alignment with current guideline standards.

**Fig. 3:**
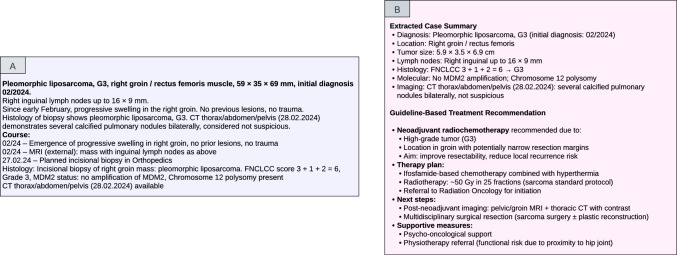
**A**, **B** Example of tumor board registration letter and ChatGPT-4o response. **A** Original tumor board registration letter used as input. **B** Corresponding output generated by ChatGPT-4o

ChatGPT (GPT-4o, OpenAI) (https://openai.com/index/hello-gpt-4o/) was used for all prompt executions. The model was accessed via its respective web interface and prompted without any fine-tuning. The prompts including tumor board registration letters were entered in German language. All test cases from the evaluation cohort were submitted sequentially within a single uninterrupted chat session, with no manual corrections or user feedback provided between runs. Figure 3A presents an example tumor board letter, while Fig. 3B shows the corresponding ChatGPT-4o output.

## Statistical analysis

No predefined sample size calculation was performed due to the retrospective and descriptive nature of the study. Scores assigned by ChatGPT were normalized to a scale from 0 to 1 by dividing the raw score of each evaluation criterion by its maximum possible value. A total normalized score was computed per case as the average across all five criteria; in cases where one criterion was missing, the total score was calculated as the mean of the available criteria. Descriptive statistics (mean, standard deviation (SD), median, interquartile range (IQR)) were computed for each criterion and for the total normalized score. Normality of the total scores was assessed using the Shapiro–Wilk test. To evaluate overall performance relative to the maximum possible score, the Wilcoxon signed-rank test was applied to compare both the total normalized score and each individual criterion against the maximum of 1.0. To test whether ChatGPT-4o performance differed across evaluation criteria, a nonparametric one-way ANOVA was performed, followed by post hoc pairwise comparisons using Dunn’s test. Multiple comparisons were adjusted using the two stage Benjamini-Krieger-Yekutieli procedure to control the false discovery rate. Differences in total normalized scores across sarcoma subtypes were assessed using the Kruskal–Wallis test. Inter-rater agreement between the two sarcoma experts was assessed using Cohen’s kappa. All analyses were two-sided with a significance level of α = 0.05. Statistical analyses were performed using GraphPad Prism (version 10.5.0).

## Results

### Study sample characteristics

A total of 1901 sarcoma cases were discussed at the institutional sarcoma tumor board between January and December 2024. From this cohort, 200 cases were randomly selected for potential inclusion. Among these, GIST and bone tumors were excluded, resulting in a preliminary cohort of 152 soft tissue sarcoma cases. One case was excluded due to incomplete documentation. Additional 13 cases were reserved for prompt development and excluded from the main analysis. The final study cohort comprised 138 patients who were presented to the multidisciplinary sarcoma tumor board for diagnostic evaluation and treatment planning. The median age of the cohort was 66 years (range: 19–88 years), including 70 male (50.7%) and 68 female (49.3%) patients.

The three most frequently represented sarcoma subtypes were leiomyosarcoma (n = 22), dedifferentiated liposarcoma (n = 16), and myxofibrosarcoma (n = 15). A detailed summary of patient demographics and histological subtypes is presented in Table 1.

**Table 1:** Demographic and clinical characteristics of the study population

Characteristics	Value
Number of patients	138
Median Age, years (range)	66 (19–88)
*Gender*	
Male sex, n (%)	70 (50.7%)
Female sex, n (%)	68 (49.3%)
*Primary Cancer*	
Leiomyosarcoma	22
Dedifferentiated Liposarcoma	16
Myxofibrosarcoma	15
Myxoid Liposarcoma	14
Synovial Sarcoma	12
Atypical Lipomatous Tumor	12
Angiosarcoma	11
Liposarcoma	6
Pleomorphic Liposarcoma	6
Solitary Fibrous Tumor	6
Undifferentiated Pleomorphic Sarcoma	7
Desmoplastic Small Round Cell Tumor	3
Malignant Peripheral Nerve Sheath Tumor	2
Pleomorphic Leiomyosarcoma	2
Rhabdomyosarcoma	2
Alveolar Soft Part Sarcoma	1
Unclear*	1

*Case presented to the multidisciplinary sarcoma tumor board without available pathological diagnosis at the time of discussion

### Inter‑rater agreement

The overall inter‑rater agreement between the two sarcoma expert reviewers was almost perfect, with a Cohen’s kappa of 0.95 across all 607 ratings. Agreement was almost perfect for Diagnostics, Therapeutic Modalities and Clinical Contextualization, each demonstrating a Cohen’s kappa value of 0.94, while perfect agreement was observed for Treatment Sequence and Timing (κ = 1.00). Chemo Regimen Selection showed substantial to almost perfect agreement, with a Cohen’s kappa value of 0.90.

### Subgroup analysis by sarcoma type

The Kruskal–Wallis test revealed no statistically significant difference in performance between sarcoma subgroups (H = 19.74, p = 0.138). Descriptive statistics of the total normalized ChatGPT-4o performance score across different sarcoma types are summarized in Table 2.

**Table 2:** Descriptive statistics of ChatGPT-4o performance across sarcoma subtypes

Sarcoma subtype	Median (IQR)*
Leiomyosarcoma	0.875 (0.771–1.000)
Dedifferentiated Liposarcoma	0.804 (0.714–0.875)
Myxofibrosarcoma	0.857 (0.750–0.857)
Myxoid Liposarcoma	0.829 (0.464–0.857)
Synovial Sarcoma	0.857 (0.750–1.000)
Atypical Lipomatous Tumor	1 (0.964–1.000)
Angiosarcoma	0.875 (0.607–1.000)
Liposarcoma	0.804 (0.616–0.964)
Pleomorphic Liposarcoma	1 (0.893–1.000)
Solitary Fibrous Tumor	0,857 (0.857–0.964)
Undifferentiated Pleomorphic Sarcoma	0.857 (0.536–0.857)
Desmoplastic Small Round Cell Tumor	1 (0.812–1.000)
Malignant Peripheral Nerve Sheath Tumor	0.938 (0.750–0.750)
Pleomorphic Leiomyosarcoma	0,75 (0.771–1.000)
Rhabdomyosarcoma	0.598 (0.585–0.612)
Alveolar Soft Part Sarcoma	0.75 (0.750–0.750)
Unclear**	0.714 (0.714–0.714)

*Interquartile range (IQR); **Case presented to the multidisciplinary sarcoma tumor board without available pathological diagnosis at the time of discussion

### Performance comparison between ChatGPT-4o recommendations and maximum achievable scores

**Fig. 4:**
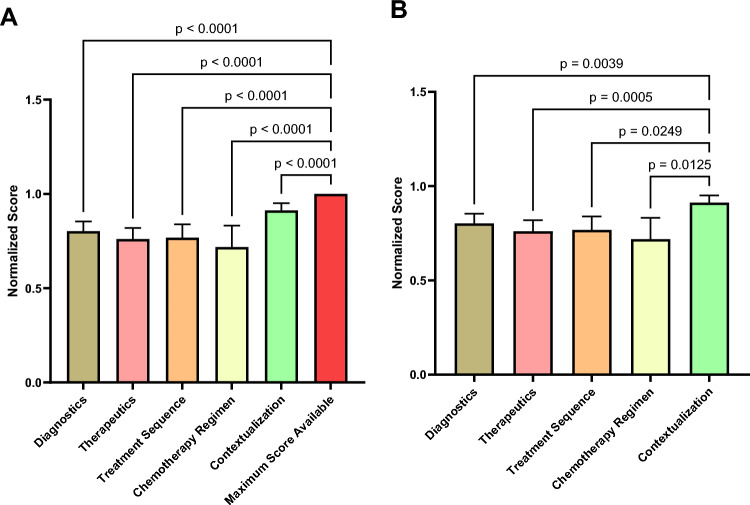
**A**, **B** Boxplots of normalized performance scores across evaluation criteria. **A** Comparison of ChatGPT-4o performance with the maximum achievable scores across all evaluation criteria. **B** Comparison of normalized performance scores across individual evaluation criteria

Shapiro–Wilk testing confirmed non-normal distribution of the total normalized scores (W = 0.830, p < 0.001). The median total normalized score across all evaluated cases was 0.857 (IQR: 0.75–1.0), with a minimum observed score of 0.125. The total normalized performance score of the ChatGPT-4o was significantly lower than the maximum achievable score of 1.0 (median < 1.0, W = 0.0, *p* < 0.0001). This finding was consistent across all five evaluation criteria, each demonstrating a statistically significant deviation from the maximum achievable score (each *p* < 0.0001). Table 3 summarizes the descriptive statistics for each criterion and the overall score, including mean ± SD, median and the distribution of assigned scores. In the corrected post hoc pairwise comparisons, Clinical Contextualization (C5) achieved significantly higher normalized scores compared to all other criteria, including Diagnostics (C1; *p* = 0.021), Therapeutic Modalities (C2; *p* = 0.001), Treatment Sequence and Timing (C3; *p* = 0.007), and Chemo Regimen Selection (C4; *p* < 0.001). No other pairwise differences reached statistical significance (all adjusted *p*-values > 0.05). Figure 4A compares the ChatGPT-4o’s scores with the maximum achievable scores, while Fig. 4B compares the scores between the individual evaluation criteria.

**Table 3:** Descriptive statistics of ChatGPT-4o performance across individual evaluation criteria and overall score

Criterion	Number of evaluated cases, n (%)	Mean ± SD*	Score = 0 (%)	Score = 1 (%)	Score = 2 (%)
Diagnostics (C1)	129 (93.5%)	0.80 ± 0.30	7 (5.4%)	37 (28.7%)	85 (65.9%)
Therapeutics (C2)	138 (100%)	0.76 ± 0.35	16 (11.6%)	34 (24.6%)	88 (63.8%)
Treatment Sequence (C3)	138 (100%)	0.79 ± 0.41	32 (23.2%)	106 (76.8%)	N/A
Chemo Regimen (C4)	64 (46.5%)	0.72 ± 0.45	18 (28.1%)	46 (71.9%)	N/A
Contextualization (C5)	138 (100%)	0.89 ± 0.24	4 (2.9%)	16 (11.6%)	118 (85.5%)
Total Normalized Score	138 (100%)	0.81 ± 0.21	N/A***	N/A	N/A

*Standard deviation; ** Interquartile range; *** Not Applicable

## Error type analysis: hallucination and confabulation

Two sarcoma experts reviewed all model outputs for error types beyond score deviations. A total of four cases were identified. Two were classified as hallucinations: one involved the incorrect mention of a translocation instead of the documented MDM2 amplification, and the other falsely assumed prior surgery, leading to a recommendation for adjuvant radiotherapy despite neoadjuvant treatment. The remaining two cases were categorized as confabulations: one involved a PET-CT recommendation not supported by guidelines, and the other misinterpreted tumor markers that were ordered due to a prior rectal carcinoma. These cases are summarized in Supplementary Table 1.

## Discussion

Large language models represent an emerging technology in artificial intelligence with potential applications in clinical decision-making and treatment recommendation generation. In oncology, where MDTs serve as the gold standard for complex treatment planning, this technology may offer supplementary support. Despite the established value of MDTs in improving clinical outcomes and guideline adherence, these collaborative platforms face significant challenges including cost constraints, geographic barriers, and potential treatment delays (Nardone et al. 2024). Consequently, exploring whether LLMs could provide supportive assistance within existing MDT workflows, while maintaining established clinical oversight, warrants investigation. In this study, we aimed to assess whether ChatGPT-4o could generate treatment recommendations for patients with STS that might align with multidisciplinary tumor board decisions.

Our results showed a high level of concordance between ChatGPT-4o and multidisciplinary tumor board recommendations. This aligns with prior research indicating that ChatGPT-4 demonstrated strong compatibility with tumor board decisions in cancer patient management (Schmidl et al. 2024). In our evaluation, clinical contextualization achieved the highest ratings among all assessed criteria, highlighting the model’s ability to integrate patient-specific factors and justify its reasoning. This finding is consistent with the study by Sorin et al. (2023c), which evaluated ChatGPT in breast cancer tumor board scenarios and reported that summarization and explanation performances were rated higher than direct clinical recommendations. This suggests that the model’s current strengths lie in interpretive and supportive rather than decisional functions. In contrast, the model showed limitations in therapeutic modalities, treatment sequence and timing, and chemotherapy regimen selection. This highlights the model’s limitations in generating precise, chronologically structured, and guideline-specific treatment pathways.. This observation closely parallels the findings by Stalp et al. (2024), who reported that while ChatGPT’s treatment recommendations were generally rated as sufficient, oncologists noted challenges particularly in sequencing treatments and maintaining precision. These consistent patterns across studies suggest that while LLMs may offer valuable support in synthesizing and contextualizing clinical information, they are currently less reliable for generating detailed and temporally ordered therapeutic strategies.

Despite its current limitations in generating detailed therapeutic plans, ChatGPT-4o may still serve as a valuable assistive tool within oncology workflows. Its strength in contextual reasoning suggests potential value during the preparation phase of tumor board discussions, where it could help synthesize patient-specific data into preliminary summaries or structured outlines (Huang et al. 2024). In resource-limited settings or low-volume centers where access to full multidisciplinary expertise is restricted, such models could assist in care planning by identifying contextual details, structuring fragmented patient histories, and drawing attention to relevant clinical information to support more informed decision-making (Tangsrivimol et al. 2025; Liu et al. 2023). Moreover, the implementation of AI in low-volume sarcoma centers may contribute to reducing disparities in sarcoma care. A recent analysis from Southwest Germany demonstrated that only 40% of sarcoma patients were treated in specialized sarcoma centers between 2019 and 2022 (Calukovic et al. 2025), despite current guidelines recommending early referral to high-volume expert centers to optimize clinical outcomes (Leitlinienregister 2025). Nonetheless, deploying LLMs in clinical environments introduces certain risks. A key issue is their tendency to produce hallucinations or confabulations, in which the model generates outputs that may sound clinically reasonable but are not actually supported by the provided data or current medical guidelines (Farquhar et al. 2024; Kalai et al. 2025). In our study, we observed occasional confabulations, including instances where ChatGPT-4o recommended PET-CT as part of the diagnostic approach, despite this modality not being endorsed by the S3-guideline for soft tissue sarcoma management (Leitlinienregister 2025). Future developments in LLM may help mitigate these limitations. One such direction involves reasoning-enhanced architectures, including models that incorporate chain-of-thought prompting or retrieval-augmented generation, are designed to produce more structured and transparent outputs by grounding responses in explicit reasoning steps and verified external knowledge sources (Li et al. 2025; Nishisako et al. 2025). Such techniques may reduce the likelihood of hallucinations and improve the consistency of guideline-based recommendations (Anh-Hoang et al. 2025; Amugongo et al. 2025). In addition, domain-specific language models trained on curated medical datasets, such as clinical guidelines and expert-labeled tumor board cases, may further enhance factual accuracy and clinical relevance (Lammert et al. 2024).

Although ChatGPT showed generally substantial performance, also in recent studies (Aubreville et al. 2025; Lam et al. 2025), current evidence does not support its autonomous use in clinical practice without expert oversight (Mu and He 2024; Beattie et al. 2025). It is important to recognize that ChatGPT-4o operates without a true clinical reasoning process. Its outputs are based on probabilistic language patterns rather than mechanistic understanding or experiential logic. Unlike human experts, the model cannot be interrogated, challenged, or refined in real time. This dynamic exchange is a core component of safe, collaborative decision-making in tumor boards. These limitations may help explain why the model struggled with treatment sequencing or chemotherapy selection, which often require nuanced judgment shaped by guideline interpretation, prior treatment response, and patient-specific constraints. Notably, for this reason, the European Society for Medical Oncology recently published guidance on the use of large language models in clinical practice, including a comprehensive literature review and consensus statements developed by a multidisciplinary group of experts (Wong et al. 2025). While using LLMs to generate clinical suggestions based on tumor board case summaries may streamline the decision-making process, this approach has inherent limitations. The use of closed-source models such as ChatGPT presents challenges related to long-term accessibility, reproducibility, and data privacy (Tangsrivimol et al. 2025; Li 2023). Moreover, relying on the model to interpret and act on structured case information may result in information loss, particularly when the input data is vague or context-dependent and cannot be reliably translated into guideline-consistent decisions (Seitl et al. 2024).

This study has several limitations that should be acknowledged. First, it was conducted at a single center, where tumor board documentation and clinical decision-making practices may differ from those at other institutions. As a result, the generalizability of our findings to broader oncology settings may be limited. Second, the tumor board letters were originally written in German and the performance of ChatGPT-4o on clinical content in non-German languages remains unexplored. Third, we did not evaluate the model across different temperature settings, which may influence the model performance and consistency of its outputs. Fourth, while the tumor board registration letters followed institutional templates, they were not fully standardized across all cases, which may have influenced how the model interpreted clinical details. Fifth, although the applied scoring system aimed to comprehensively reflect the key decision-making dimensions of the tumor board, it may not fully capture the nuanced rationale and interdisciplinary deliberations that occur during live MDT discussions. Sixth, ChatGPT-4o is a closed-source model, which limits transparency, reproducibility, and long-term accessibility. Finally, selection bias cannot be excluded, as cases with incomplete documentation or missing tumor board records were excluded from the study cohort.

In conclusion, ChatGPT-4o demonstrated substantial expert-rated performance in generating tumor board recommendations in patients with soft tissue sarcomas. Its greatest strength lies in personalized contextualization, where it consistently integrates case-specific details into plausible recommendations. While its diagnostic and therapeutic suggestions were often appropriate, notable discrepancies were observed, particularly in treatment sequencing and chemotherapy regimen selection. These findings highlight the potential of large language models as supportive tools in oncology workflows but also underscore the importance of human oversight. Further development and clinical validation will be essential to ensure safe, reliable, and ethically responsible integration of LLMs into real-world decision-making. In addition, research into domain-specialized language models, particularly those trained for medical applications, is warranted to evaluate their suitability for supporting multidisciplinary oncology workflows.

## Supplementary Information

Below is the link to the electronic supplementary material.

Supplementary Material 1

## Data Availability

The datasets generated and analysed during the current study are available from the corresponding author on reasonable request.

## Abbreviations

STS: Soft tissue sarcoma
MDT: Multidisciplinary Tumor Board
AI: Artificial intelligence
NLP: Natural language processing
LLM: Large language model
GPT: Generative pretrained transformer
GIST: Gastrointestinal stromal tumors
