# Supplementary material for: When AI joins the table: evaluating large language model performance in soft tissue sarcoma tumor board decisions
Source: J Cancer Res Clin Oncol. 2026 Feb 27;152(2):52. doi: 10.1007/s00432-026-06432-w (PMC12948744; doi:10.1007/s00432-026-06432-w)
Supplement: Supplementary file 1 — Supplementary Material 1 [file 432_2026_6432_MOESM1_ESM.docx]

| *Supplementary Table 1: Identified Cases of Hallucination and Confabulation Errors in Model Output.* | | | | | | | | | | | | | | |
| --- | --- | --- | --- | --- | --- | --- | --- | --- | --- | --- | --- | --- | --- | --- |
|  |  |  |  |  |  |  |  |  |  |  |  |  |  |  |

| **Type** | **Case description** |
| --- | --- |
| Confabulation | Tumor markers (CEA and CA 19-9) were within normal limits and were therefore considered by the model to be non-informative for disease monitoring in this case of leiomyosarcoma. However, it is important to note that these markers were obtained due to the patient’s prior history of rectal cancer and were not intended for the assessment or follow-up of the sarcoma. |
| Confabulation | The model recommends PET‑CT imaging, although PET‑CT is not recommended in the relevant sarcoma guidelines for this clinical scenario. |
| Hallucination | The model incorrectly reports the presence of a translocation, whereas the documented molecular finding is an MDM2 amplification. |
| Hallucination | The model falsely assumes that surgical resection has already occurred and recommends adjuvant radiotherapy, despite neoadjuvant radiotherapy having been performed and surgery still being pending. |
